# Posterior fossa ependymoma in childhood: 60 years event-free survival after partial resection—a case report

**DOI:** 10.1007/s00381-015-2766-7

**Published:** 2015-06-06

**Authors:** Tryggve Lundar, Bernt Johan Due-Tønnessen, Bård Krossnes, Paulina Due-Tønnessen, Petter Brandal

**Affiliations:** Department of Neurosurgery, Oslo University Hospital, Postboks 4950 Nydalen, 0424 Oslo, Norway; Department of Pathology, Oslo University Hospital, Oslo, Norway; Department of Oncology, Oslo University Hospital, Oslo, Norway; Department of Radiology, Oslo University Hospital, Oslo, Norway

**Keywords:** Posterior fossa ependymoma, Partial resection, Pediatric neurosurgery, Very long-term follow-up

## Abstract

A 13-year-old boy with severe clinical symptoms and signs underwent surgery for a posterior fossa ependymoma in 1954. The tumor was adjacent to the floor of the fourth ventricle, and surgery was complicated by profound bleeding. Therefore, only a partial resection was performed. Postoperative radiotherapy was given to the posterior fossa. The recovery was uneventful, and he has been in full-time work until the age of 62 years and is now 74 years old. Repeated MRI scans demonstrate a stable residual fourth ventricular tumor.

## Introduction

Ependymoma is the third most common posterior fossa tumor in childhood after astrocytoma and medulloblastoma/PNET [1, 2]. A spinal primary location is exceedingly rare in children, as opposed to adults, where a spinal presentation is the most common of all ependymoma locations [5]. Although there is anecdotal evidence that some patients can be successfully treated with surgery only following gross total resection (GTR), the role of postoperative radiation has clearly been established in retrospective studies and prospective protocols. As a consequence, the current management of intracranial ependymomas is based on maximal surgery followed by conformal radiation to the tumor bed. So far, the role of chemotherapy has not been established [3]. The importance of complete tumor resection in ependymoma is critical. Several series have documented the negative impact of incomplete resection on survival. Despite the use of radiation, the outcome of these patients with residual tumor is dismal and the current recommendation is to consider repeat resection when residual tumor is present on postoperative imaging prior to initiation of radiation treatment. Although incomplete resection in patients with posterior fossa ependymoma usually anticipates a negative outcome, there may be some exceptions to this rule, as illustrated in the following case report.

## Case report

In September 1954, a 13-year-old boy was referred to our department with vertigo, episodes of unclear vision, and later on, vomiting and headache. Clinical investigation revealed choked disks with hemorrhages, nystagmus, a slight right-sided facial paresis, dysdiadochokinesis, and spasticity in the right arm as well as a positive Babinski sign on the left side.

Ventriculography demonstrated an enlarged supratentorial ventricular system and a tumor in the fourth ventricle. The patient had surgery where a partial occipital bone resection was performed with opening of the foramen magnum and laminectomy of atlas. The dura was pulseless, but after removal of 60 cm^3^ CSF from the lateral ventricle, the intradural pressure was normalized. After dural incision, a red-brown tumor was exposed medially in the right cerebellar hemisphere and into the fourth ventricle. The tumor was resected, but only partially removed because it was adjacent to the floor of the fourth ventricle, and surgery was complicated by the occurrence of profound bleeding. Microscopic examination revealed a well-circumscribed, moderately cellular glial tumor with several pseudorosettes. No mitoses, microvascular proliferation, or necrosis was found. The tumor cells were positive for glial fibrillary acid protein (GFAP) and negative for epithelial membrane antigen (EMA), synaptophysin, neurofilament protein, and the IDH-1 (R132H mutation). Less than 5 % of the tumor cells were Ki-67 positive. The tumor was classified as an ependymoma, WHO grade II.

Postoperatively, the boy recovered clinically within weeks and the choked disks resolved completely. During the next 2 months, he was given high-voltage radiotherapy to the posterior fossa (45 Gy in 30 fractions).

Later on, he returned to school and finished a year later than his peers. He married and got two children. After 26 years as a bank officer, the started his own accountant firm.

**Fig. 1:**
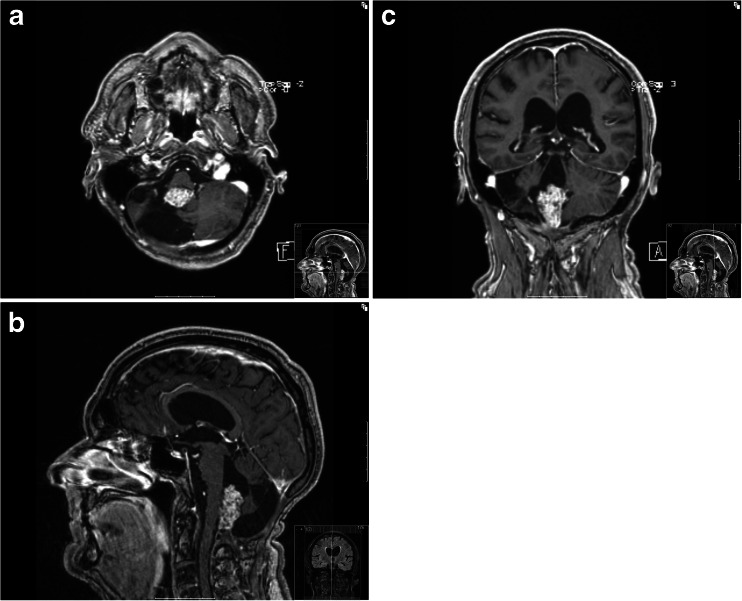
MRI of the posterior fossa discloses contrast-enhancing tumor in the fourth ventricle. The situation has been stable for several years, and the patient is without clinical symptoms and signs

He worked full time until the age of 62. At the age of 67, he experienced an episode of syncope but recovered completely without treatment. An MRI demonstrated a large contrast-enhancing tumor of the fourth ventricle. His clinical situation has been stable and good, and the MRI findings are also unchanged after several years of observation (Fig. 1).

## Discussion

Long-term survival after operation for posterior fossa ependymoma in childhood has been observed for many years, especially after GTR [4, 7–9, 11]. Tumor free survival has even been observed after repeat surgery due to recurrence [11]. Because of the high risk of tumor recurrence, postoperative radiotherapy has been implemented to the posterior fossa and, in earlier years also, to the craniospinal axis. The latter has been abandoned because local control seems to be the key to cure [6]. Due to the harmful effects of CNS radiation in small children, such treatment has often been postponed under the age of 3 years and, in some centers, below the age of 5 years [9].

In small children, chemotherapy has been given as a substitute, but the benefit remains uncertain.

**Fig. 2:**
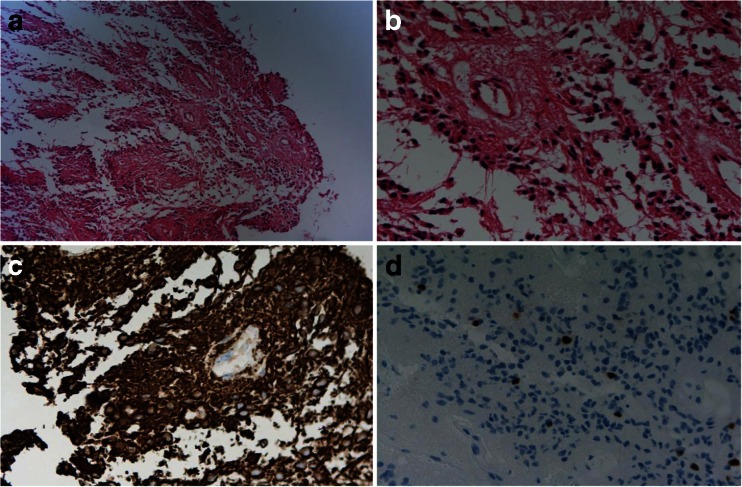
Pathological features of the tumor. A glial tumor with pseudorosettes was seen (**a**, **b**). The tumor cells were positive for GFAP (**c**). Less than 5 % of the tumor cells were Ki-67 positive (**d**)

The present case demonstrates that a very good clinical result may follow after initial surgery, even when incomplete, when followed by local radiation. This is in contrast to our previous experience, and such a good result is not to be expected. The original pathology has been reviewed and confirms the diagnosis of an ependymoma, WHO grade II (Fig. 2).

This now 74-year-old man has no clinical symptoms from the posterior fossa, and 3 different MRI studies have not demonstrated any progression with respect to his residual tumor during a 4-year period. The first MRI was taken after an episode of syncope, 54 years after primary treatment. Most probably, his residual tumor has been present ever since the operation in 1954. The situation therefore seems stable and resembles the puzzling situation we have observed in a small group of pediatric patients with recurrent disease decades after primary treatment for distal spinal ependymoma [5].

In 2011, Witt and colleagues described two clinically and molecularly distinct subgroups of posterior fossa ependymoma [12]. Group A patients, with a more serious prognosis, had lower age and lateral tumor position in the posterior fossa, while group B patients were older with midline tumors. Our patient, therefore, very likely could represent a group B ependymoma with an indolent behavior.

The present case is also in accordance with the statement made by van Veelen-Vincent and colleagues, “The risk of recurrence or progression becomes quite small once the first 5 years after the initial surgery have passed uneventfully, regardless of whether initial surgery was complete or incomplete or whether the patient received radio- or chemotherapy” [9].

Many children who survive treatment for a posterior fossa ependymoma are left with permanent neurological sequelae after aggressive surgical resection and may furthermore suffer from long-term consequences of radio- or chemotherapy [10]. The quality of life for long-term survivors is a challenging and important topic that is sometimes underreported when compared to survival results. In the patient reported here, the surgical resection was only partial due to tumor infiltration in the floor of the fourth ventricle as well as intraoperative hemorrhage, and radiotherapy to the posterior fossa was administered.

Although the prognosis was considered dubious, the boy recovered completely. It is therefore important to notice that a remarkably good result may occur even for a very long time even if a GTR is not achieved.
